# Supplementary figures and images for: Lower respiratory tract microbiota in patients with clinically suspected nontuberculous mycobacterial pulmonary disease according to the presence of gastroesophageal reflux
Source: PLoS One. 2024 Aug 28;19(8):e0309446. doi: 10.1371/journal.pone.0309446 (PMC11355550; doi:10.1371/journal.pone.0309446)

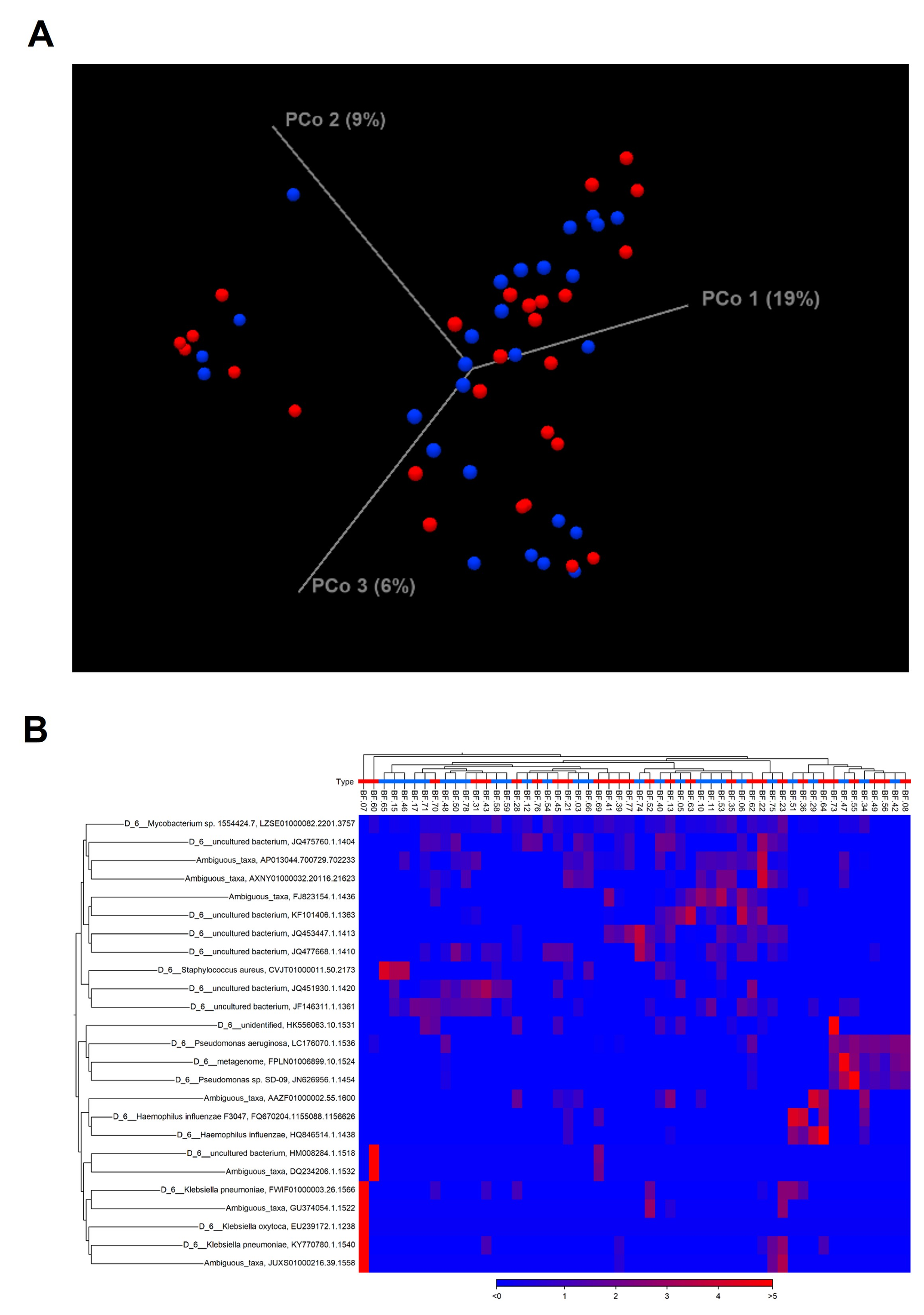

Supplement: S1 Fig — There was no significant difference in the composition of the lower lung microbiome between bacteriologically confirmed NTM-PD (blue dots) and non-NTM-PD group (red dots) (A). Unweighted beta diversity analysis showed the overall bacterial community structure and phylogenetic diversity for bacteriologically confirmed NTM-PD group (blue) and non-NTM-PD group (red)(B). (TIF) [file pone.0309446.s001.tif]

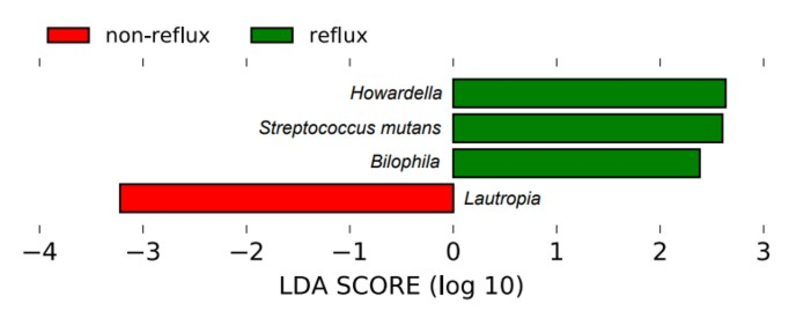

Supplement: S2 Fig — The length denotes the effect size for a taxon. P = 0.05 for the Kruskal-Wallis test; LDA score >2.5. (TIF) [file pone.0309446.s002.tif]

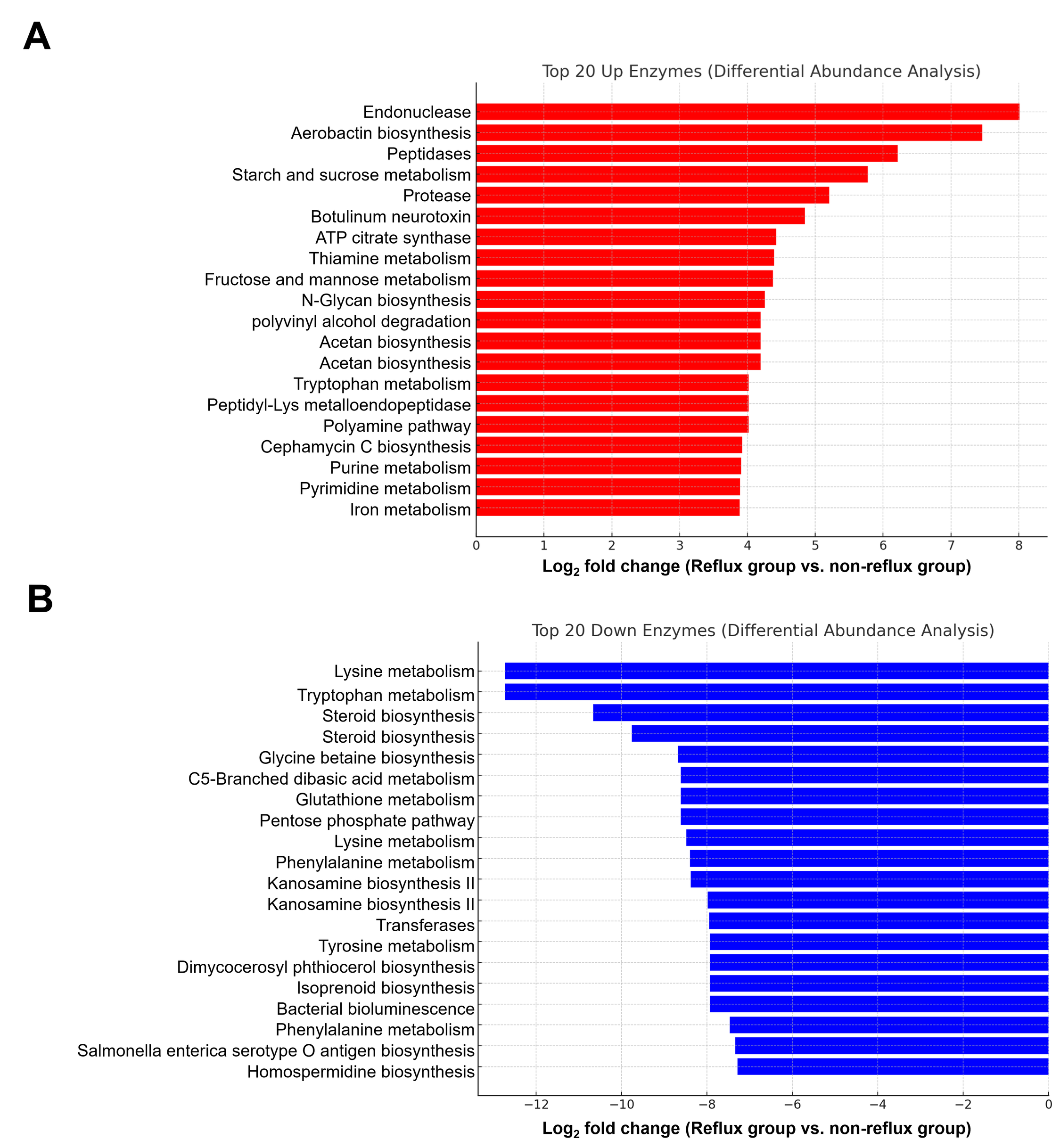

Supplement: S3 Fig — Several functional categories related to aerobactin biosynthesis, nucleic acid/protein/iron metabolism, energy production, or other cellular metabolism were up-regulated in reflux group (A). To the contrary, categories related to antioxidant defense or production of steroid hormones were down-regulated, as such lysine metabolism and steroid biosynthesis in reflux group (B). (TIF) [file pone.0309446.s003.tif]
