# Supplementary material for: Lower respiratory tract microbiota in patients with clinically suspected nontuberculous mycobacterial pulmonary disease according to the presence of gastroesophageal reflux
Source: PLoS One. 2024 Aug 28;19(8):e0309446. doi: 10.1371/journal.pone.0309446 (PMC11355550; doi:10.1371/journal.pone.0309446)
Supplement: S1 Table — (DOCX) [file pone.0309446.s004.docx]

**Table S1. Baseline characteristics of the patients enrolled in this study**

|  | **Total (n=47)** | **NTM-PD (n=24)** | **Non-NTM-PD (n=23)** | ***P*-value** |
| --- | --- | --- | --- | --- |
| **Age, median (IQR)** | 63.0 (56.0-74.0) | 66.5 (56.8-76.5) | 62.0 (56.0-74.0) | 0.277 |
| **Sex (male), n (%)** | 17 (36.2) | 6 (25.0) | 11 (47.8) | 0.135 |
| **Ever smoking, n (%)** | 11 (23.4) | 3 (12.5) | 8 (34.8) | 0.093 |
| **Previous tuberculosis, n (%)** | 7 (14.9) | 4 (16.7) | 3 (13.0) | 1.000 |
| **Diabetes, n (%)** | 4 (8.5) | 3 (12.5) | 1 (4.3) | 0.609 |
| **Heart disease, n (%)** | 5 (10.6) | 2 (8.3) | 3 (13.0) | 0.666 |
| **Chronic kidney disease, n (%)** | 2 (4.3) | 0 (0) | 2 (8.7) | 0.234 |
| **GERD, n (%)** | 22 (46.8) | 12 (50.0) | 10 (43.5) | 0.772 |

Abbreviations: GERD, gastroesophageal reflux disease; IQR, Interquartile range; NTM, non-tuberculosis mycobacterium; PD, pulmonary disease.
