# Supplementary material for: Lower respiratory tract microbiota in patients with clinically suspected nontuberculous mycobacterial pulmonary disease according to the presence of gastroesophageal reflux
Source: PLoS One. 2024 Aug 28;19(8):e0309446. doi: 10.1371/journal.pone.0309446 (PMC11355550; doi:10.1371/journal.pone.0309446)
Supplement: Attachment — Submitted filename: PlosONE_reviewer answer file 0510.docx [file pone.0309446.s005.docx]

Academic Editor
PLOS ONE

Journal Requirements:

When submitting your revision, we need you to address these additional requirements.

1. Please ensure that your manuscript meets PLOS ONE's style requirements, including those for file naming. The PLOS ONE style templates can be found at

https://journals.plos.org/plosone/s/file?id=wjVg/PLOSOne_formatting_sample_main_body.pdf and

https://journals.plos.org/plosone/s/file?id=ba62/PLOSOne_formatting_sample_title_authors_affiliations.pdf.

Answer: We have modified a revised manuscript according to the PLOSONE’s style.

2. We noticed you have some minor occurrence of overlapping text with the following previous publication(s), which needs to be addressed:

https://err.ersjournals.com/content/errev/30/160/200299.full.pdf?

https://www.mdpi.com/2079-6382/10/7/766?

In your revision ensure you cite all your sources (including your own works), and quote or rephrase any duplicated text outside the methods section. Further consideration is dependent on these concerns being addressed.

Answer: We have re-checked a revised manuscript as recommendation.

3. Thank you for stating in your Funding Statement:

“This study was supported by grants from the National Research Foundation of Korea (NRF) Grant funded by the Ministry of Education, Science, and Technology (grant no. NRF- 2022R1C1C1002741 (Eun Jeong Won) and 2020R1F1A1076570 (Yong-Soo Kwon)). The funding body played no role in the design of the study and collection, analysis, interpretation of data, and in writing the manuscript.”

Please provide an amended statement that declares *all* the funding or sources of support (whether external or internal to your organization) received during this study, as detailed online in our guide for authors at http://journals.plos.org/plosone/s/submit-now.  Please also include the statement “There was no additional external funding received for this study.” in your updated Funding Statement.

Please include your amended Funding Statement within your cover letter. We will change the online submission form on your behalf.

Answer: We have added Funding Statement within cover letter as recommendation.

4. We note that you have included the phrase “data not shown” in your manuscript. Unfortunately, this does not meet our data sharing requirements. PLOS does not permit references to inaccessible data. We require that authors provide all relevant data within the paper, Supporting Information files, or in an acceptable, public repository. Please add a citation to support this phrase or upload the data that corresponds with these findings to a stable repository (such as Figshare or Dryad) and provide and URLs, DOIs, or accession numbers that may be used to access these data. Or, if the data are not a core part of the research being presented in your study, we ask that you remove the phrase that refers to these data.

Answer: We have added Supporting information (Table S1).

5. Please be informed that funding information should not appear in the Acknowledgments section or other areas of your manuscript. We will only publish funding information present in the Funding Statement section of the online submission form. Please remove any funding-related text from the manuscript.

Answer: We have removed Funding information in the Acknowledgments as recommendation.

6. Your ethics statement should only appear in the Methods section of your manuscript. If your ethics statement is written in any section besides the Methods, please move it to the Methods section and delete it from any other section. Please ensure that your ethics statement is included in your manuscript, as the ethics statement entered into the online submission form will not be published alongside your manuscript.

Answer: We have added ethics statement in the Methods section as recommendation.

7. We notice that your supplementary figures are included in the manuscript file. Please remove them and upload them with the file type 'Supporting Information'. Please ensure that each Supporting Information file has a legend listed in the manuscript after the references list.

Answer: We have added Supporting information as recommendation.

[Note: HTML markup is below. Please do not edit.]

Reviewers' comments:

Reviewer's Responses to Questions

**Comments to the Author**

1. Is the manuscript technically sound, and do the data support the conclusions?

The manuscript must describe a technically sound piece of scientific research with data that supports the conclusions. Experiments must have been conducted rigorously, with appropriate controls, replication, and sample sizes. The conclusions must be drawn appropriately based on the data presented.

Reviewer #1: Yes

Reviewer #2: Partly

Reviewer #3: Yes

2. Has the statistical analysis been performed appropriately and rigorously?

Reviewer #1: Yes

Reviewer #2: Yes

Reviewer #3: Yes

3. Have the authors made all data underlying the findings in their manuscript fully available?

The PLOS Data policy requires authors to make all data underlying the findings described in their manuscript fully available without restriction, with rare exception (please refer to the Data Availability Statement in the manuscript PDF file). The data should be provided as part of the manuscript or its supporting information, or deposited to a public repository. For example, in addition to summary statistics, the data points behind means, medians and variance measures should be available. If there are restrictions on publicly sharing data—e.g. participant privacy or use of data from a third party—those must be specified.

Reviewer #1: Yes

Reviewer #2: Yes

Reviewer #3: Yes

4. Is the manuscript presented in an intelligible fashion and written in standard English?

PLOS ONE does not copyedit accepted manuscripts, so the language in submitted articles must be clear, correct, and unambiguous. Any typographical or grammatical errors should be corrected at revision, so please note any specific errors here.

Reviewer #1: Yes

Reviewer #2: Yes

Reviewer #3: Yes

5. Review Comments to the Author

Please use the space provided to explain your answers to the questions above. You may also include additional comments for the author, including concerns about dual publication, research ethics, or publication ethics. (Please upload your review as an attachment if it exceeds 20,000 characters)

Reviewer #1: This paper illustrated the microbial characteristics in the lower respiratory tract due to reflux in NTM-PD patients. While it may not play a direct role in clinical treatment, I believe it is an extremely important study for understanding the pathophysiology of NTM-PD.

Q1) [Line 89-99 "typical findings of the disease on the chest CT] It would be beneficial to provide additional details on the aspects described in lines 89-99.

Answer: We added the typical findings of chest CT as follow (Lines 99-101). “characterized by bilateral multilobar bronchiectasis and small nodules predominantly observed in the right middle lobe and the lingular segment of the left upper lobe”

Q2) [Line 105 "BAL samples] Were BAL samples adequately collected from all patients (retrieve volume and color)? Were there any samples that did not pass quality control due to low biomass? Was there significant variation in valid read values? How was normalization carried out for read count and gene copy number?

Answer: During bronchoscopy, BAL samples were collected by instilling 30 mL of 0.9% NaCl three times into the lung at a subsegmental level, where the lesions appeared to have the most active infection with micronodules and bronchiectasis. The first and third fractions of specimens were sent for a diagnostic microbiological evaluation as part of standard care. Second fraction of specimen was immediately transferred onto ice to lab. Samples were promptly spun at 10,000 g, within 30 minutes of BAL sample collection. The cell pellets in 1 mL were stored at −80°C . DNA was extracted and then was sent to Macrogen for 16S rRNA sequencing. During the two steps of library quality control, 23 samples were excluded because of a low quantity of DNA extracted from specimens. Finally, 47 cases were included for this microbiome study. Mean (SD) value of total reads of each sample was 63,453 (16, 418) reads, and there was no significant variation among 47 samples included in this study. We have described about our procedure in detail in revised manuscript (Lines 106-112; 129).

Q3) [Line 157 "24 patients were bacteriologically confirmed NTM-PD] Were these patients confirmed to have NTM through BAL fluid culture?

Answer: Yes. All 24 patients tested positive for NTM culture in BAL fluid specimens


Q4) [Line 166 Table, NTM-PD with reflux] Shouldn't "NTM-PD with reflux" in Table 1 be accurately labeled as "suspected NTM-PD with reflux"?

Answer: We have modified labeling within Table 1.

Q5) [Line 188-190] It would be advisable to mention in the limitations that Pseudomonas aeruginosa can also be found in conditions of severe lung parenchymal damage (not just reflux). Were there any clinical differences between NTM-PD patients without reflux and those with reflux? (e.g., extent of lung involvement or bacterial load).

Answer: Thank you for your valuable comments. We agreed that that *Pseudomonas aeruginosa* is commonly associated with structural lung diseases such as bronchiectasis, potentially leading to lung parenchymal damage. Therefore, patients with reflux may exhibit more severe lung involvement and a higher prevalence of *P. aeruginosa* colonization. Our analysis categorized the extent of lung involvement into mild, moderate, and severe based on the number of affected lobes and the presence of bilateral disease or cavities in chest CT scans. Among our patient cohort, 25 individuals (53.2%) exhibited mild disease, while 22 (26.8%) showed moderate disease; no severe cases were recorded. Notably, patients with reflux were more likely to present with moderate disease compared to those without reflux (17 [77.3%] vs. 5 [20.0%], p = 0.001), indicating a greater extent of lung involvement in this subgroup. However, it remains unclear whether this difference in disease severity correlates with alterations in the microbiome, posing a potential limitation to our study. We added it in the Discussion section (Lines 274-285).

Reviewer #2: 16s gene amplicon sequencing to characterize the microbiota of lower respiratory and GI tract from clinical samples. Patients suspected NTM-PD were selected and included cohorts were NTM-PD was confirmed by bacteriological assay, as well as individuals without confirmation. The samples were further divided between those with and without reflux. Bronchoscopy samples were used for DNA extraction and amplicon sequencing. While no significant difference in overall diversity among patients with suspected NTM-PD, lower diversity was observed in the reflux group. Specific bacterial taxa were found to be associated with efflux.

Despite the potential importance of the questions being addressed, the study is lacking in the level of analysis typically seen for studies assessing microbiota structure. Specific points include:

1. Since 16s gene amplicon sequencing was used to identify bacterial taxa, the microbiome, which includes genes and genomes, was not assessed. References to the microbiome should be changed to “microbiota”.

Answer: We have changed references as reviewer’s recommendation.

2. Unfortunately, no LRT samples recovered from bronchoscopy not associated with NTM-PD were analyzed. The lung microbiota should be considered from a greater diversity of samples, even if data from public repositories are analyzed.

Answer: We have added above limitations in revised manuscript as reviewer’s recommendation (Lines 287-288; 297-301).


3. Beyond bacterial taxa, the authors should identify pathways/functions that correlate with the clinical characteristics. This can be accomplished using standard bioinformatic tools.

Answer: We entirely agree the need to further identify the pathways/functions that correlate with the clinical characteristics. Although this provisional study could not add aforementioned data, our data could support the need to large-scale study with tunned scheme for elucidating this notion. We have added above limitations in revised manuscript according to the reviewer’s comments (Lines 297-301).


4. Fig. 3 claims to show the composition of the lower lung microbiota at the species level. This is placing too much confidence in the results of 16s rRNA gene amplicon sequencing and the figure should be adjusted to show more accurate taxonomic assignments.

Answer: The number of listing of taxonomic assignments may appear somewhat arbitrary. We aimed to present this figure in a way that it could infer the composition of the lower lung microbiota between two groups. Therefore, we believe that the top 15 taxonomic assignments provide sufficient insight into their composition patterns in majority. If it is acceptable, we would prefer to keep the current figure unchanged.

5. If reflux is a mechanism for “seeding” the lung with microorganisms from the gut,

Answer: Thank you for your insightful comment. Reflux has the potential to alter the microbiome by either seeding microbes from the stomach or modifying the respiratory tract environment through the aspiration of gastric contents. However, we could not explain the precise mechanisms underlying these microbiome changes in patients with reflux in this study (Lines 239-254).

6. The limited conclusions from the work do not consider the biology of the bacteria whose differences are noted. A more rigorous assessment of how the bacterial taxa could be relevant. For example Fig. 4 lists strict anaerobic bacteria typically associated with the gut microbiota. What are the implications of these observations?

Answer: We have more discussed about the biology of the bacteria whose differences were noted, such as anaerobic bacteria associated with the gut microbiota, in revised manuscript (Lines 239-254).

Reviewer #3: This is a well-written paper and addresses an important topic in NTM-LD. I have a few concerns which are as follows:
1) The introduction needs a hypothesis and summary of results.

Answer: We have already delineated the hypothesis of this study concerning the variances in the microbiome's composition in the presence of GERD (Lines 69-91).

2) The authors have chosen all subjects with NTM-LD. This means the cohort must be very diverse. Some patients would have bronchiectasis and others would not. This needs to be discussed and in particular how this must be affecting the microbiome.

Answer: We have added above limitations in revised manuscript as reviewer’s recommendation (Lines 286-303).

3) There are no immunological corrollaries in this study. This is a limitation and therefore must be discussed. The paper is descriptive which is fine but should be discussed as a limitation.

Answer: We have added above limitations in revised manuscript as reviewer’s recommendation (Lines 299-303).

6. PLOS authors have the option to publish the peer review history of their article (what does this mean?). If published, this will include your full peer review and any attached files.


If you choose “no”, your identity will remain anonymous but your review may still be made public.


**Do you want your identity to be public for this peer review?** For information about this choice, including consent withdrawal, please see our Privacy Policy.

Reviewer #1: No

Reviewer #2: No

Reviewer #3: No

[NOTE: If reviewer comments were submitted as an attachment file, they will be attached to this email and accessible via the submission site. Please log into your account, locate the manuscript record, and check for the action link "View Attachments". If this link does not appear, there are no attachment files.]

While revising your submission, please upload your figure files to the Preflight Analysis and Conversion Engine (PACE) digital diagnostic tool, https://pacev2.apexcovantage.com/. PACE helps ensure that figures meet PLOS requirements. To use PACE, you must first register as a user. Registration is free. Then, login and navigate to the UPLOAD tab, where you will find detailed instructions on how to use the tool. If you encounter any issues or have any questions when using PACE, please email PLOS at figures@plos.org. Please note that Supporting Information files do not need this step.
