# Supplementary material for: Lower respiratory tract microbiota in patients with clinically suspected nontuberculous mycobacterial pulmonary disease according to the presence of gastroesophageal reflux
Source: PLoS One. 2024 Aug 28;19(8):e0309446. doi: 10.1371/journal.pone.0309446 (PMC11355550; doi:10.1371/journal.pone.0309446)
Supplement: Attachment — Submitted filename: PlosONE_reviewer answer file R2.docx [file pone.0309446.s006.docx]

2024-07-20

Dear Editor,

Thank you very much for your review and for returning our manuscript for revision. Enclosed please find a file that contains the revised manuscript entitled “*Lower respiratory tract microbiota in patients with clinically suspected nontuberculous mycobacterial pulmonary disease according to the presence of gastroesophageal reflux*”, as well as itemized responses to the reviewer’s comments. We highlighted the changes to this manuscript within the document by using blue colored text.

We look forward to any additional comments or questions concerning this paper and hope that the revised manuscript is now acceptable for publication in PLOS ONE.

Sincerely,

Yong-Soo Kwon, M.D., Ph.D., on behalf of all the authors.

Department of Internal Medicine

Chonnam National University Hospital

Gwangju, Korea

E-mail: yskwon@jnu.ac.kr

Reviewer number two suggested a more comprehensive analysis of the data to identify features of the samples that are more informative than bacterial taxa alone. For example, PICRUSt (now PICRUSt2, DOI: 10.1038/s41587-020-0548-6), which predicts functional potential of a bacterial community based on marker gene sequencing profiles, is a standard tool for analyzing 16s amplicon sequencing data. The continued lack of marker gene analysis leaves the reader lacking potentially useful information that can better inform future studies.
Answer: We have added PICRUSt data as S3 Figure and we have described functional data with discussion in revised manuscript accordingly (Lines 151-152; 217-224; 298-304). We have added more references and rearranged them accordingly.

The following misstatements should be corrected:

1. 16S ribosomal RNA sequencing was not done, rather 16S ribosomal RNA gene amplicon sequencing was performed.
Answer: We have corrected the word throughout manuscript as recommendation.

2. 16s amplicon sequencing is used to interrogate the microbiota, not the microbiome, as specific genes and genome are not being characterized. This was noted by original reviewer #2 but was not changed in the title, keywords and abstract.

Answer: We have revised the word throughout manuscript as recommendation.

3. The abstract states that: “These observations confirm that the lower respiratory microbiome is reliably altered by reflux but not in NTM-PD”. The word “reliably” is awkward and should be replaced with “consistently”.

Answer: We have corrected the word as recommendation (Line 59).
